# Gene Signatures Derived from a c-MET-Driven Liver Cancer Mouse Model Predict Survival of Patients with Hepatocellular Carcinoma

**DOI:** 10.1371/journal.pone.0024582

**Published:** 2011-09-16

**Authors:** Irena Ivanovska, Chunsheng Zhang, Angela M. Liu, Kwong F. Wong, Nikki P. Lee, Patrick Lewis, Ulrike Philippar, Dimple Bansal, Carolyn Buser, Martin Scott, Mao Mao, Ronnie T. P. Poon, Sheung Tat Fan, Michele A. Cleary, John M. Luk, Hongyue Dai

**Affiliations:** 1 Rosetta Inpharmatics LLC, Merck & Co., Inc., Seattle, Washington, United States of America; 2 Department of Surgery, The University of Hong Kong, Pokfulam, Hong Kong, China; 3 Department of Pharmacology, Department of Surgery, and Cancer Science Institute, National University of Singapore, Singapore, Singapore; 4 Merck Research Laboratories, Merck & Co., Inc., Boston, Massachusetts, United States of America; 5 Molecular Profiling and Pharmacology, Merck & Co., Inc., North Wales, Pennsylvania, United States of America; Institute of Molecular and Cell Biology, Singapore

## Abstract

Biomarkers derived from gene expression profiling data may have a high false-positive rate and must be rigorously validated using independent clinical data sets, which are not always available. Although animal model systems could provide alternative data sets to formulate hypotheses and limit the number of signatures to be tested in clinical samples, the predictive power of such an approach is not yet proven. The present study aims to analyze the molecular signatures of liver cancer in a c-MET-transgenic mouse model and investigate its prognostic relevance to human hepatocellular carcinoma (HCC). Tissue samples were obtained from tumor (TU), adjacent non-tumor (AN) and distant normal (DN) liver in Tet-operator regulated (TRE) human c-MET transgenic mice (n = 21) as well as from a Chinese cohort of 272 HBV- and 9 HCV-associated HCC patients. Whole genome microarray expression profiling was conducted in Affymetrix gene expression chips, and prognostic significances of gene expression signatures were evaluated across the two species. Our data revealed parallels between mouse and human liver tumors, including down-regulation of metabolic pathways and up-regulation of cell cycle processes. The mouse tumors were most similar to a subset of patient samples characterized by activation of the Wnt pathway, but distinctive in the p53 pathway signals. Of potential clinical utility, we identified a set of genes that were down regulated in both mouse tumors and human HCC having significant predictive power on overall and disease-free survival, which were highly enriched for metabolic functions. In conclusions, this study provides evidence that a disease model can serve as a possible platform for generating hypotheses to be tested in human tissues and highlights an efficient method for generating biomarker signatures before extensive clinical trials have been initiated.

## Introduction

Hepatocellular carcinoma (HCC) is the fifth most common malignancy worldwide, with over 300,000 new cases per year in China and with a rising incidence in western countries [1]. Surgical resection or liver transplantation are the primary treatment options for HCC patients having a 5-year survival rate at 50–60% [2]. Unfortunately, about 80% of patients are diagnosed in advanced stages at presentation and are essentially inoperable and refractory to most of the conventional chemotherapies [3]. As such, there is an urgent need to identify prognostic markers of HCC [4], [5], [6], [7], [8], [9] and to develop targeted therapies through conventional small molecule inhibitors and/or RNAi therapeutics [10], [11], [12], [13], [14].

Several intricate transgenic mouse models of human cancer have been suggested to accurately mimic the pathophysiology and molecular features of human malignancies [15], but cross-species gene-expression comparisons of the animal models and human disease are not available for validation [16]. HCC develops in humans as a progressive disease from a cirrhosis predisposition caused by hepatitis B or C virus infection, chronic alcoholism, or aflatoxin exposure. As a result, human HCC tumor tissue is surrounded by premalignant cirrhotic tissue [17]. A transgenic mouse model of HCC has been developed by Bishop and colleagues in which tumors are induced by liver-specific, tetracycline-regulated (TRE) expression of a human c-MET kinase transgene, a genetic lesion commonly associated with human liver tumors [18]. The tumors that arise due to c-MET over-expression in the mouse resemble human HCC at the level of histology [19]. Activating mutations in β-catenin leading to upregulation of the Wnt signaling pathway, another common feature of human HCC, were frequently observed in these tumors. Nevertheless, information on tumor suppressor gene TP53, which is commonly mutated in human HCC [20], and other potential gene targets in this model system are not available. Furthermore, the molecular character of the adjacent non-malignant tissue surrounding the tumors is not well studied and characterized [21]. A better understanding of how the mouse model compares with human disease at the molecular level is therefore crucial to the design and interpretation of efficacy studies for therapies.

Biomarkers derived from microarray expression profiling data can be subject to high false-positive rate due to multiple hypothesis testing inherent to working with large numbers of genes and gene combinations. A predictive biomarker signature or gene set determined from a given set of samples (the training set) must be validated with data from independent samples (the test/validation set) [22], [23]. Meeting this goal can be challenging as independent data sets, especially those from clinical samples treated in a similar manner, are scanty or require significant time investment to accumulate. One work-around to this limitation is to formulate and test hypotheses using data from a model system.

In this study, we performed molecular profiling of normal liver and tumor tissues from the c-MET driven mouse model, to understand the molecular changes in these mice. We determined how well the model approximates human disease and confirmed the expression of specific cancer targets. We used the data derived from the c-MET model to generate signatures distinguishing tumor (TU) from adjacent non-tumor (AN) and wild-type (WT) normal tissues, and tested the prognostic power of these signatures in a data set from human HCC.

## Methods

### Ethics

The Institutional Review Board of the University of Hong Kong/Hospital Authority Hong Kong West Cluster (HKU/HA HKW IRB) approved this study, and each patient gave his/her written informed consent on the use of the clinical specimens for research. All studies involving animals were fully approved by the Merck Boston Institutional Animal Care and Use Committee (protocol numbers: #07-08-044 and #08-08-041) and were conducted according to the institutional animal ethics guidelines.

### The c-MET mouse HCC model

The mice used in this study have been described (Table 1) [18], [24]. All mice were on an FVB genetic background. Mice overexpressing human c-MET carried one copy of the LAP-tTa transgene (the liver-specific LAP promoter driving the Tet-VP16 transactivator) and one copy of the TRE-c-MET transgene (Tet-operator regulated human c-MET gene). The presence of both transgenes results in expression of the human c-MET gene specifically in and throughout the liver (referred to henceforth as the TRE-c-MET strain). Seven mice of each strain were sacrificed at six (TRE-c-MET), seven (LAP-tTa) or 14 (TRE-c-MET) weeks of age. Normal liver or liver tumor tissue (two per mouse) was collected and processed for gene expression profiling at the Rosetta Gene Expression Laboratory. In addition, adjacent liver tissue was collected from the non-involved tissue next to the border of the tumor in the tumor-bearing liver lobe. Distant liver tissue was from a non-tumor bearing lobe or from areas at least 1 cm away from the tumor. Animal works were conducted in AALAC-accredited laboratory according to the institutional animal ethics guidelines.

**Table 1:** Mouse signatures identify gene sets with predictive power for survival in human samples.

	Tumor	
	Down(p-value)	Up(p-value)
**WT**	9.5×10^−6^	0.07
**AN**	2.0×10^−5^	0.10
**DN**	2.3×10^−5^	0.06

Pair-wise comparison between tumors and wild type (WT), adjacent non-tumor (AN) or distant normal (DN) liver samples identified expression signatures for genes that were either down-regulated or up-regulated in the tumors. The p-values for the ability of these signatures to predict survival in human patients is indicated based on K-M curves in Figure 3.

### Patient cohorts and clinical samples

All patients that were enrolled in this study underwent a curative hepatectomy for HCC at Queen Mary Hospital, Pokfulam, Hong Kong between 1993 and 2007 [3], [25]. This study was approved by the Institutional Review Board for Human Ethics and each patient gave his/her written informed consent on the use of the clinical specimens for research. Liver tissue that was obtained from patients at the time of the curative surgery was immediately snap-frozen in liquid nitrogen and stored at −80°C until required.

### Microarray and analysis

Total RNA was extracted and purified from the clinical liver specimens (n = 272 HBV-HCC tumor (TU), 257 HBV-HCC tumor-adjacent normal (AN), 9 HCV-HCC tumor (TU).

9 HCV-HCC tumor-adjacent normal (AN)) using the SV96 Total RNA Isolation System (Promega) according to a custom automated protocol. The extracted RNA was quantified using RiboGreen RNA Quantitation Reagent (Invitrogen) and its quality was assessed using Agilent RNA 6000 Pico Kit (Agilent, Santa Clara, CA) in an Agilent 2100 Bioanalyzer (Agilent). Only those samples passing the minimum thresholds for quantity and quality (RIN>6) were amplified and labeled using the Ovation WB protocol (NuGEN Technologies, San Carlos, CA), according to the manufacturer's instructions. In brief, 50 ng of total RNA was amplified using the Ribo-SPIA technology (NuGEN Technologies),fragmented and labeled with biotin using the FL-Ovation cDNA Biotin Module V2 (NuGEN Technologies). The resulting amplified cRNAs were hybridized to Affymetrix gene expression chips (Human Rosetta Custom Affymetrix 1.0, Affymetrix, Santa Clara, CA) [26]. The images were analyzed using the standard package of Affymetrix GeneChip Operating Software (GCOS) (www.affymetrix.com/products/software/specific/gcos.affx) and were further normalized and processed to derive the sequence-based intensities using the RMA algorithm as implemented in Affymetrix Power Tools (http://www.affymetrix.com/support/developer/powertools). Data used for this analysis passed two levels of quality controls (QCs) (array level using Affymetrix recommended parameters, and project level on excluding outlier arrays and arrays with major patterns associated with known process parameters). Log10(ratio) of each gene in each sample were computed by subtracting mean of log10(Intensity) of that gene across all adjacent non-tumor samples, to make them comparable to the c-MET mouse model data where the references are the pool of wild-type mouse liver tissues. Raw gene expression profiling data were deposited to GEO with the following accession numbers: GSE25142 (derived from the c-Met mouse model) and GSE25097 (derived from human HCC).

### Tumor signature from mouse profiles

We used one-way ANOVA to define tumor signature in mice c-MET experiment, say, for a comparison between WT Vs. tumor, we identified 6277 mouse probesets with ANOVA P-value < 0.001. The false discovery rate (FDR) here is estimated to be 0.62% from 1000 permutations. Among those signature probesets, 3114 showed tumor down- and 3163 tumor up-regulations compared with WT. The mouse tumor signature was then mapped to human probesets in Affymetrix human chip. We also identified mouse tumor signature from comparisons of adjacent non-tumor vs. tumor and distant non-tumor vs. tumor, by ANOVA analysis.

### Biological annotation and geneset enrichment test

We compiled many databases include gene sets with known biological functions or properties from a variety of public (GO cellular components, molecular function, biological processes, KEGG pathways, SwissProt Keywoards, etc.), licensed (GeneGo, Ingenuity, NextBio biosets, etc.), and proprietary sources (internal compound, siRNA, body atlas profiling, etc.). These annotated gene sets were used in the enrichment test. The enrichment P-values (the chance probability of observing overlapping genes between the input geneset and the geneset in the database) is computed using the hypergeometric distribution [27].

### Prognostic power of signatures

To estimate the prognostic power of each of mouse signatures, the signature was mapped to human probesets and then treated as a metagene [28], [29]. Namely, the expression level for the metagene in human HCC samples was calculated by averaging log(ratio) of all the genes mapped from the mouse up- or down-regulated signatures. The human HCC samples were ranked by the expression level of the metagene and then divided into two equal groups by the median value (to avoid the over-fitting, we did not optimize the threshold). The log-rank P-values between these two groups were calculated by the log-rank test using time of overall survival and disease free survival respectively.

## Results

### Global gene expression changes in mouse and human HCC

**Figure 1:**
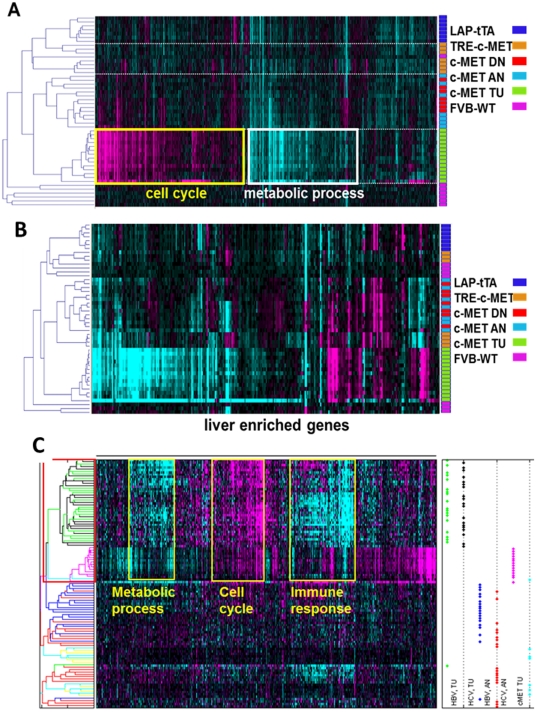
Molecular profiling of the mouse c-MET HCC tumor model. (A) Global gene expression analysis revealed tumor-specific gene expression changes characterized by genes down-regulated in tumors that were enriched for metabolic processes (white box) and genes up-regulated in tumors that were enriched for cell cycle and actin cytoskeleton (yellow). Non-tumor tissues adjacent to or distant from the tumors showed similar expression patterns are were interspersed in the heat map generated by unsupervised clustering, with samples from the same animal clustering together. The vertical bars to the right of each heat map represent color coding of the samples that corresponds to the legend in each panel. (FVB-WT, purple) Liver tissue from control animals; (LAP-tTA, dark blue and TRE-c-MET, orange) liver tissue from single transgene parental strains; (c-MET TU, green) tumor tissue from double transgene, tumor bearing animals; (c-MET AN, light blue) non-tumor liver tissue from double transgene, tumor-bearing animals adjacent to tumor; (c-MET DN, red) non-tumor liver tissue from double transgene, tumor-bearing animals distant from tumor. The heat map represents unsupervised clustering of differentially-expressed genes (fold change ≥1.25, p<0.01, Cluster Algorithm: Agglomerative, Similarity Measure: Cosine correlation). (B) The majority of liver-enriched genes were down-regulated in the c-MET tumors, consistent with loss of liver function. For this analysis, we selected the top 400 genes with the greatest fold-change from the liver-enriched genes identified by Su and colleagues [18]. A small subset of genes that were up-regulated (yellow boxes) may contain useful biomarkers of tumor presence. Color legend −0.5<log_10_ (ratio)<0.5. (C) Comparison of mouse c-MET and human HCC tumor profiles. Human and mouse tumor samples co-clustered indicating similar gene expression patterns (red box). The differentially expressed genes can be divided into three groups on the basis of their specific regulation patterns (yellow boxes) and GO annotation. (HCC Adj) Human adjacent non-tumor; (HCC TU) human tumor; (c-MET TU) mouse tumor; (c-MET Adj/Dis) mouse adjacent and distant normal. The heat map represents unsupervised clustering of differentially-expressed genes (fold change ≥1.25, p<0.01, Cluster Algorithm: Agglomerative, Similarity Measure: Cosine correlation).

To determine tumor-specific gene expression in the c-MET mouse model of HCC, we compared tissue from tumor-bearing mice to several control tissues including adjacent and distant normal liver tissue from tumor bearing mice, wild-type liver tissue and liver tissue from two single transgene parental lines (Table S1). We used wild-type liver tissue (virtual pool from 7 mice) as a baseline and performed unsupervised clustering of differentially-expressed genes. We found that the tumors had a distinct expression pattern (Figure 1A). To characterize the molecular nature of the differentially expressed genes, we performed Gene Ontology biological annotation [30] (see also Supplementary Information) on each gene set and found that the down-regulated genes were enriched for metabolic processes, whereas the up-regulated genes were enriched for cell cycle and cytoskeleton-related terms (Table S2). Gene expression changes in human HCC showed similar GO annotations (Table S3), indicating that on a global gene expression level, the c-MET mouse model approximates human HCC.

We performed unsupervised clustering and found that the expression profiles of adjacent and distant normal tissue samples were interspersed, segregated by the animal from which they were taken and distinct from the tumor profiles (Figure 1A). This result indicates that tumor proximity does not significantly alter gene expression in the normal liver tissue. However, subtle differences between the adjacent and distant samples may exist.

Although both HCV- and HBV-infection have been shown to cause HCC, HBV infections were predominant in this cohort (272 HBV-HCC tumors and 9 HCV-HCC tumors). To identify potential molecular differences between HCV- and HBV-infected HCC, we analyzed all available HCV samples in this cohort (nine) and compared them to an equal number of randomly selected HBV samples (nine). The HBV samples were randomly distributed in an unsupervised cluster of all tumor samples (data not shown) indicating that they were not skewed toward a particular molecular profile. We did not observe any consistent molecular difference among these samples (data not shown).

To determine whether parallels between human and mouse HCC exist at the gene level, we performed direct comparison of the mouse and human HCC profiles. The mouse samples were normalized against wild-type liver tissue (virtual pool from 7 mice) and the human samples were normalized against an average of all adjacent non-tumor samples. Unsupervised clustering of the human and mouse samples showed that the molecular profiles of the two tumor sets were more closely related to each other than to their cognate adjacent non-tumor tissues (Figure 1B, red box), revealing a tumor-specific molecular signature.

Among the genes differentially expressed in the majority of mouse and human tumors, we identified three gene subsets with distinct characteristics (Figure 1B, yellow boxes). Two groups showed similar expression patterns in both tumor types: genes down-regulated in both tumor types were enriched for metabolic processes, whereas genes up-regulated in both tumor types were enriched for cell cycle processes. Genes down-regulated specifically in the human tumors were enriched for immune response processes, reflecting a molecular distinction that may point to differences in tumor progression mechanisms. In summary, comparison of the molecular profiles of human and mouse HCC revealed extensive parallels at the gene expression level. However, each set of tumors was also characterized by specific gene expression patterns.

Down-regulation of genes involved in metabolic processes in the tumors suggests that liver functions are diminished or impaired. To explore liver identity in these tumors further, we examined the expression of liver-enriched genes [31], [32], [33] and found that they were down-regulated in the tumor samples (Figure 1C), consistent with loss of liver identity and function with tumor progression.

### Activity of oncology pathways in HCC

**Figure 2:**
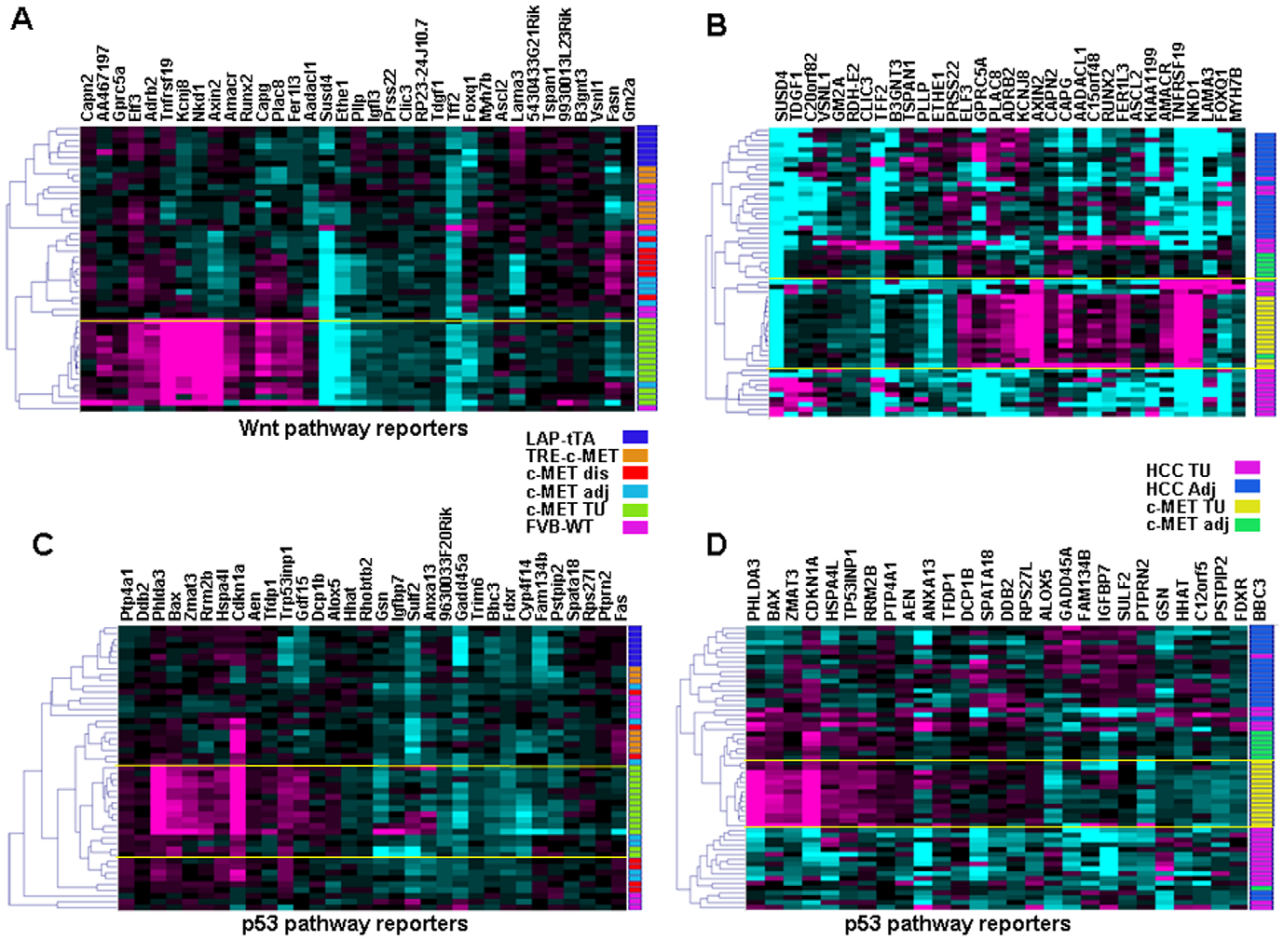
Activity of oncogenic pathways in mouse c-MET tumors and HCC. (A) The Wnt/β-catenin pathway was up-regulated in the mouse tumors as measured by the Wnt/β-catenin gene signature [34]. The genes in each signature are indicated across the top of each heat map. The samples are on the Y-axis and their tissue of origin is indicated in the vertical color-coded bar to the right of each heat map. Color-coding legend for panels A and C is between the panels. All abbreviations are as in Figure 1. (B) The Wnt/β-catenin pathway was up-regulated in a subset of human HCC patients as illustrated by the three human samples (HCC TU, purple) that co-cluster with the mouse HCC samples (c-MET TU, yellow). Color-coding legend for panels B and D is between the panels. (C) The tp53 pathway was up-regulated in the mouse tumors(c-MET TU, green). (D) Up-regulation of the tp53 pathway was specific to the mouse model (c-MET TU, yellow) and was not observed in human HCC (HCC TU, purple).

To gain a better understanding of the activity of signaling pathways relevant to oncology in the c-MET model of HCC, we performed targeted analysis of the expression changes using pathway signatures defined previously. The Wnt/β-catenin signature consists of genes up- and down-regulated by β-catenin siRNAs in DLD-1 colon carcinoma cells [34]. We found elevated Wnt pathway activity (Figure 2A), consistent with the activating mutations of β-catenin frequently detected in these tumors and activation of the Wnt pathway in one third of HCCs [35]. Unsupervised clustering of the mouse and human samples showed a relationship between a subset of human HCC profiles and the mouse tumor samples (Figure 2B), indicating that the c-MET mouse may be a useful model for studying HCC patients with activated Wnt signaling. Interestingly, the genome-wide profiles of the mouse tumors and the subset of human HCC with up-regulated Wnt pathway expression were not correlated (correlation = 0.07). This is in contrast to the significant correlation observed for the focused Wnt signaling pathway gene set, indicating that the similarities are restricted to specific pathways.

p53 pathway activity in the mouse HCC model is of interest as *TP53* mutations are common (in ∼27% of cases) in human HCC [20]. We used a p53 pathway signature [36] and observed up-regulation of the p53 pathway in the mouse HCC tumors, demonstrating a difference from human HCC (Figure 2C). In contrast, human HCC samples did not show p53 pathway up-regulation (Figure 2D), presumably due to mutations or other p53-inactivating mechanisms. These results indicate that although the mouse c-MET model may replicate a subset of human HCCs in several aspects, molecular differences do exist and should be taken into account when data obtained from this model are analyzed, particularly for targets in the p53 pathway. This is largely due to the wild-type status of p53 gene in the mouse model. Thus, special attention and consideration should be paid when comparing the cross-species human disease model. The Wnt signaling pathway and the p53 pathway are all very common in cancer development. Gene expression signatures of both pathways in the mouse tumor model and human HCC were presented in Figure S2.

### Mouse-derived gene signatures have predictive power for survival of human HCC patients

We identified gene signatures in the mouse tumors by comparing the tumor gene expression pattern to the three non-tumor tissues indicated in Table S1 (wild type (WT), adjacent nontumor (AN) and distant normal (DN) liver). We used the adjacent vs. tumor signature because it is most analogous to the comparison of the clinical samples in our study. The distant vs. tumor signature provides further information about any effects that tumor proximity may exert on the adjacent tissue. Finally, given that both the adjacent and the distant tissues also express the c-MET transgene, we included the wild-type vs. tumor signature to identify any c-MET-driven gene expression changes.

**Figure 3:**
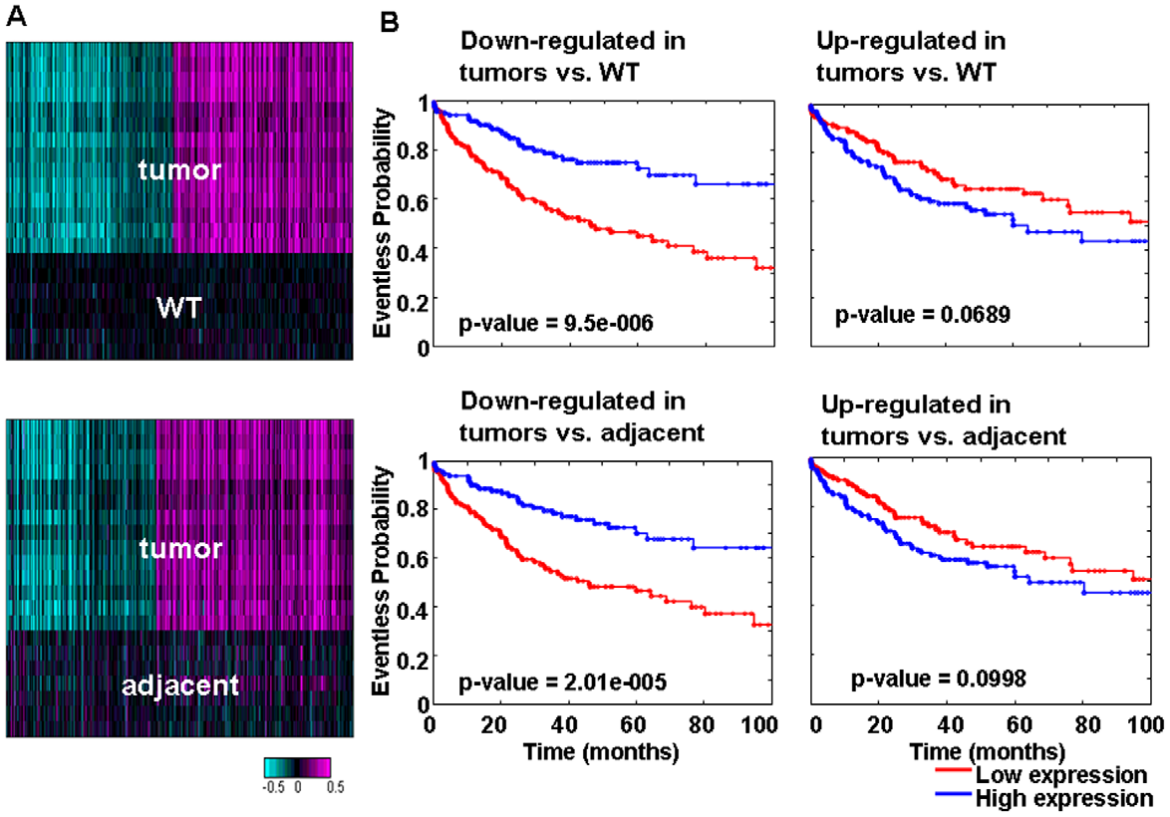
Mouse tumor signatures predict human patient survival. (A) Mouse tumor gene expression signatures. Heat maps show the expression of genes that were differentially expressed between tumor tissue and wild type or adjacent normal tissues in the mouse. (B) The mouse tumor signatures were split into up-regulated and down-regulated sets and Kaplan-Meier plots were generated for each gene set to test the predictive power for overall patient survival.

For each pair-wise tissue comparison, we identified sets of genes that were down- or up-regulated in the tumor and generated heat maps using these genes and the mouse c-MET tumor and FVB-WT wild-type samples shown in Figure 3A and Figure S1. We then projected those signatures to the human HCC data and determined their survival predictive power and their expression pattern. The entire cohort of patients was used in this analysis and the human samples were divided into two groups based on the average log(ratio) of all genes in the signature as described in the Materials and Methods.

Interestingly, we found that for all comparisons, the genes down-regulated in the tumors had highly predictive power for patient survival (Table 1) and disease free survival (Table S4). As shown previously, these genes were enriched for metabolic processes both in humans and mouse. In contrast, the up-regulated signature, enriched for cell cycle processes, did not have very high predictive power for survival (Table 1) or disease free survival (Table S4). Figure 3B and Figure S1 show the Kaplan-Meier plots for these data. We suggest that the up-regulated cell cycle processes lack predictive power because they represent general tumor events, whereas loss of metabolic properties signifies specific loss of functional properties by the liver cells that may be detrimental to patient survival.

To determine whether the predictive power of the mouse-derived signatures is specific to the c-MET model, we analyzed the predictive power of a signature derived from independent mouse models [37] and found that a significant portion of the genes had predictive power (Figure S3). These results indicate that the predictive power of the mouse-derived genes comes from the tumor properties of the mouse samples and suggests a general utility of mouse tumor models for identification of gene signatures predictive of outcome in human tumors.

**Figure 4:**
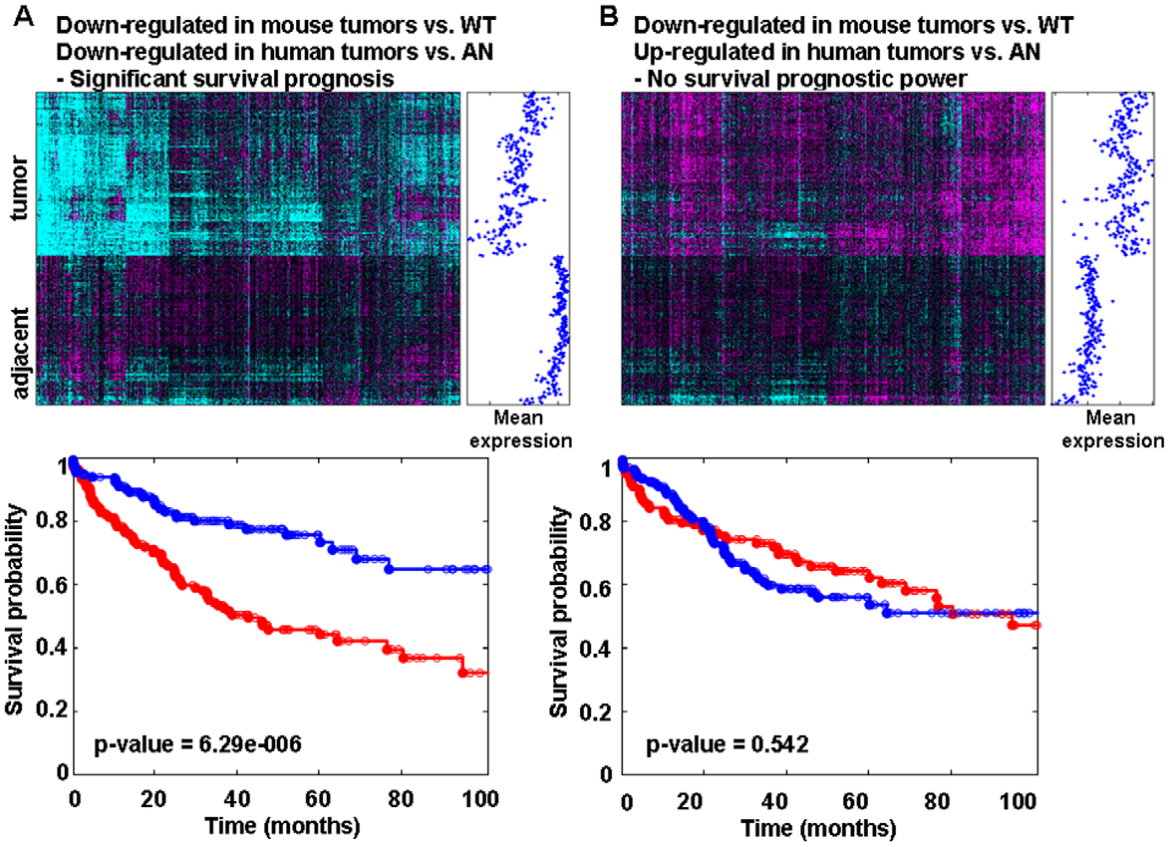
Expression of mouse signature genes in human tumors. Gene signatures generated in mouse tissues were projected onto the human HKU HCC data set. The color scale is as in Figure 1 (−0.5 to 0.5). Mean expression levels are plotted to the right of each heat map to illustrate the association between expression and prognosis. The K-M curves are given below each heat map.

Next, we analyzed the expression pattern of the mouse gene signatures in the human samples and found that the genes identified in the mouse model showed significant expression changes in the human tumors (representative heat maps in Figure 4.) To determine whether the expression changes were in the same direction in the mouse and human, we calculated the average expression for each gene in the human tumors compared with adjacent non-tumor. We found that each of the six mouse signatures contained genes whose expression changed in the human tumors both in the same and in the opposite direction. For example, among the genes down-regulated in mouse tumors vs. WT tissue (Figure 4A–B), a subset was also down-regulated in human tumors (Figure 4A) whereas a subset was up-regulated in human tumors compared to adjacent non-tumor tissues (Figure 4B). Similarly, among the genes that were up-regulated in mouse tumors vs. WT tissue, a subset of genes was down-regulated in the human tumors and a subset was up-regulated (data not shown).

To understand the difference between the genes regulated in the same or in the opposite direction in the mouse and human tumors, we analyzed each subset separately for their power to predict survival (Table 2), disease-free survival (Table S5) and for enrichment for biological pathways (Table 2). We found that the genes down-regulated both in mouse and human tumors (Table S6) retained a highly significant predictive power for survival and enrichment for metabolic processes. In contrast, the subset of genes that were down-regulated in the mouse tumors but up-regulated in human tumors did not have predictive power and significant enrichment for any biological processes. Among the genes up-regulated in the mouse tumors, those that were down-regulated in the human tumors did not have any predictive power for survival or significant biological annotation (significant enrichment of biological pathways, as measured by hypergeometric P-value). Interestingly, whereas the entire set of genes up-regulated in the mouse tumors did not have a predictive power (see above, Table 1 and Table S4), the subset of genes that were up-regulated in both mouse and human tumors showed marginally significant predictive power (Table 2, Table S7). This result indicates that filtering out the discordantly regulated genes through a model system and clinical samples and retaining only those that are similarly regulated in both can reveal sets with predictive power that may not be detected when either the global signatures of either system are considered separately.

**Table 2:** Prognostic power and GO Biological process annotation of mouse signatures split according to their expression in human samples.

Mouse	Down in tumor		Up in tumor	
Human	down	up	down	up
**Tumor compared to**				
WT	6.3×10^−6^	0.54	0.32	0.021
adjacent	2.7×10^−6^	0.66	0.51	0.0026
distant	1.1×10^−5^	0.58	0.45	0.0089
WT	metabolism	no enrichment	cell motility	cell cycle
adjacent	metabolism	no enrichment	no enrichment	cell cycle
distant	metabolism	no enrichment	cell motility	cell cycle

Mouse signatures were split according to the expression of the genes in human tissues. The ability of each gene set to predict overall survival in human samples was assessed using K-M plots. The p-values for prognosis were calculated and are indicated in the table. Each gene set was analyzed for enrichment of gene ontology biological pathways.

Since the mouse HCCs were induced by c-MET, we repeated the above analysis in human HCC by focusing on those patients with high c-MET (patients with HCC c-MET expression > median expression of the population) to define the signatures in the same or opposite direction between mouse and human. Similar to Figure 4 A&B, we identified 775 genes both down regulated in mouse and human c-MET-high HCC, and 612 genes in the opposite direction. Among these, 749 overlapped with 800 same direction genes using all HCC samples (96.7% overlap, hypergeometric P-value 0) and 562 overlapped with 587 opposite direction genes using all HCC samples (95.7% overlap, hypergeometric P-value 0). Analogues to Figure 4 and Table 2, we also checked the prognostic power of these two signatures, the log-rank P-values for overall survival are 2.2×10−6 and 0.78 respectively, very similar predictive power as the case where the whole HCC patients were used to map the overlap signatures (Table 2). Gene ontology of these overlapping genes was conducted to reveal the biological pathways associated with different gene sets when comparing the mouse c-MET driven liver tumors and the human c-MET-high HCC (Table S8).

## Discussion

The present study shows that the mouse c-MET tumor model has similarities with human HCC at the molecular level including down-regulation of metabolic processes and up-regulation of cell cycle genes. Tumor-specific gene signatures derived in the mouse model can distinguish tumor from non-tumor tissue in human HCC. The genes down-regulated in tumor compared with adjacent non-tumor tissue in both the mouse and human samples had significant predictive power on overall survival and disease-free survival in HCC patients. These genes were highly enriched for metabolic function indicating that loss of normal liver function is related to poor outcome in HCC patients. The predictive power of the mouse-derived signatures likely stems from their tumor properties rather than c-MET-driven properties, and underscores the utility of mouse tumor models for identification of gene signatures relevant to human disease.

**Figure 5:**
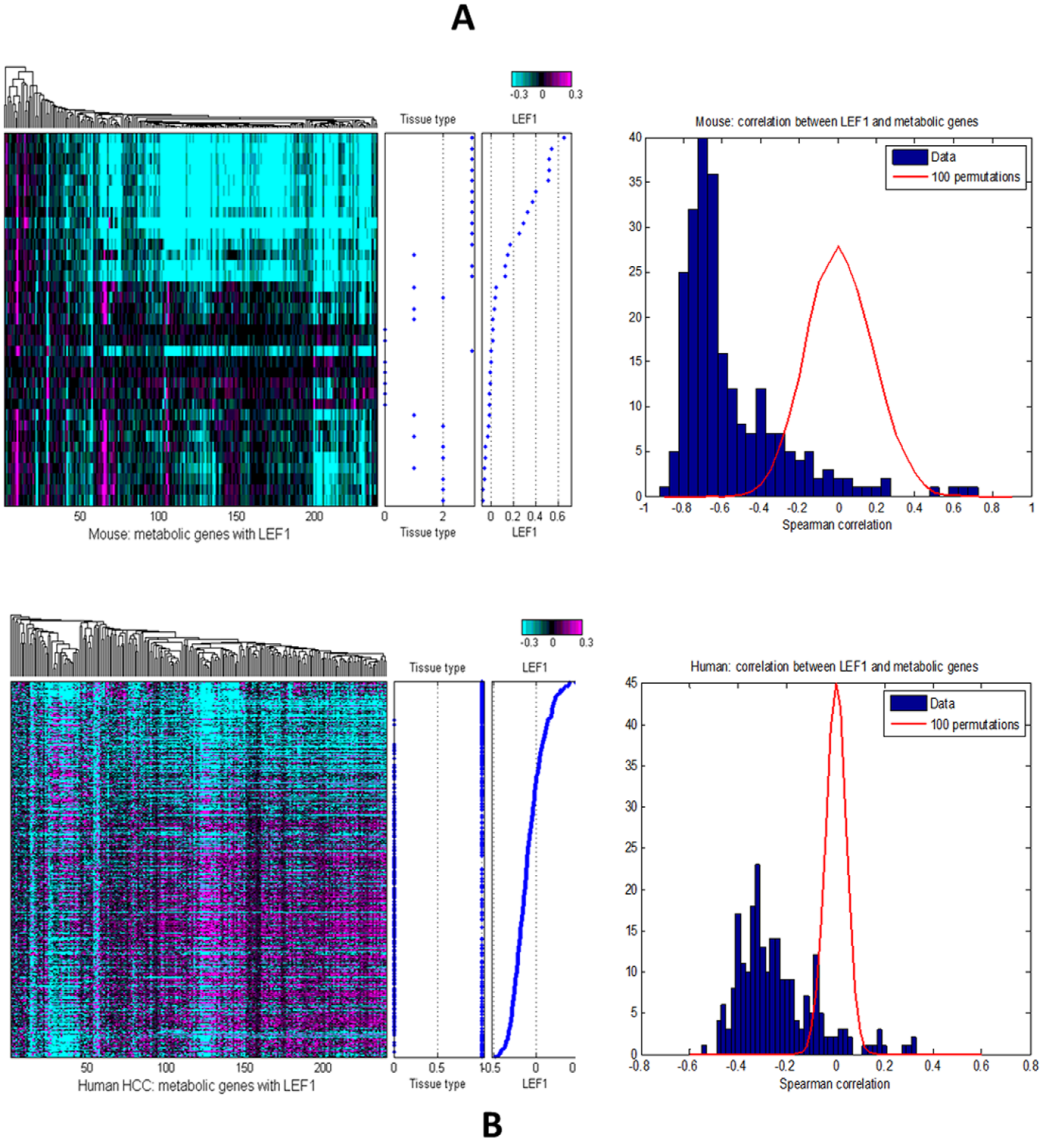
Anti-correlation of metabolic gene signatures with Wnt pathway gene LEF1. Heat map of 250 metabolic genes and their correlation with LEF1 expression level in (A) mouse c-MET liver tumors and (B) human HCC. Log rank test p-value tested in mouse and human HCC samples for metabolic genes derived by LEF1 compared with permutation. Spearman's test was used to calculate the correlation. Mouse tissue types: 0 = WT, 1 = DN, 2 = AN, 3 = TU; human tissue types: 0 = AN; 1 = TU.

The tumors from the c-MET model had uniform gene expression profiles as expected for tumors induced by a single oncogene in an inbred mouse strain. This is in contrast with human HCC samples, which showed significant differences in oncology pathway activity and mRNA expression [38], [39]. Significantly, by comparing the mouse and human expression profiles, we found that the mouse model is similar to a subset of human tumors characterized by high levels of Wnt pathway activity. Given that Wnt activation is a unique pattern activated in c-MET induced HCC and that the downregulated metabolic genes in mouse had prognostic power in human HCC, we examined the correlation between Wnt activation and metabolic dysfunction in both mouse and human HCC samples. We found that metabolic function was anti-correlated with activation of the Wnt pathway both in animal model and human HCC (**Figure 5**). The trend is stronger in mouse than in human, which may indicate that more factors affect metabolic function in human tumors.

Human cancers are thought to initiate from a single mutated cell in the context of a normal organ, whereas in the c-MET mouse model, all liver cells overexpress the oncogene, potentially creating a different microenvironment for the pre-neoplastic cell. The mechanism by which only certain cells within the c-MET-overexpressing liver develop into cancer is unclear at present, although the presence of a secondary mutation is necessary. We compared the gene signatures from c-MET overexpressing non-tumor tissues (adjacent or distant) to signatures from liver tissue of wild-type mice and did not find any biological annotation or predictive power (data not shown). The similar predictive power and biological annotations of the signatures regardless of the control tissue used in the comparison (wild type, adjacent or distant) suggests that, on a global level, gene expression changes in the tumor are minimally influenced by c-MET overexpression and, for the most part, reflect downstream consequences of tumor formation. In support of this hypothesis, the c-MET-regulated genes identified in primary hepatocytes from the c-MET knock-out mouse [40] did not show significant regulation in the c-MET overexpressing non-tumor tissues (data not shown). Within our own human HCC dataset, we found little differences in terms of mouse and human overlapping down-regulated or up-regulated tumor to normal signatures no matter whether we used the whole HCC populations or the subpopulation with higher c-MET expression. The independence of the tumor signature on c-MET expression indicates that the tumor signature is not a consequence of the model-specific tumor-initiating lesion. Rather, the tumor signature reflects downstream effects of tumor progression that are analogous in the mouse model and in human disease. These findings are significant because they underscore the relevance of the mouse model to human HCC despite the inherent difficulties in recapitulating human tumor initiation in the mouse.

With respect to human disease, we found that the down-regulated signature in tumor vs. adjacent non-tumor tissue that is shared by mouse and human samples has very significant predictive power on overall survival and disease free survival. Further refinement of this signature may identify genes that can be tested as predictive biomarkers in the clinic. The down-regulated genes in tumor samples are highly enriched for metabolic function indicating that loss of normal liver function is related to poor outcome in HCC patients who received surgical treatment. Our recent study also identified down regulation of microRNA-122 significantly impaired liver mitochondrial metabolic functions in HCC [41]. In contrast, only the portion of the mouse up-regulated signature that was also up-regulated in human samples had any, albeit minimal, predictive power. As the full mouse up-regulated signature and the subset that is also up-regulated in humans are enriched for cell cycle processes, we propose that the predictive power of this set may stem from fundamental properties of the human tumors, which vary in aggressiveness and presumably the level of expression of cell cycle genes, compared with the c-MET-driven mouse tumors, which should be homogeneous.

We observed the greatest difference between human HCC and the mouse c-MET model with respect to the activity of the p53 pathway. In human HCC, the p53 pathway is frequently inactivated due to inherited or sporadic mutations in *TP53*. Activation of the p53 pathway in the mouse tumors may be part of a stress response caused by overexpression of the c-MET oncogene. Inactivation of the p53 pathway in the c-MET mouse model or overexpression of c-MET in a *TP53* mutant mouse may generate a mouse model that is more highly representative of human HCC.

Our results highlight the potential value of investing in molecular profiling of animal models of human disease. The approach described here is widely applicable to a variety of diseases for which both relevant animal models and clinical samples are readily available. Furthermore, from biomarker development point of view, any predictive or prognostic biomarkers need to go through two stages: biomarker identification (or hypothesis generation), and biomarker validation (hypothesis testing). This usually requires at least two independent datasets: training and validation set. Without appropriate validation set, due to the high dimensional nature (more than thousands of genes or signatures) of the microarray platform, signatures derived from the training set are subjective to over fitting or false positives (a property of multi-testing). Ideally this is done by two independent clinical cohorts. However, in many cases they are not readily available. This is especially true for new treatments entering the early clinical phases (Phase I & II). The HCC example described above shows it's possible to use the mouse model as the first step for signatures (and hypotheses) generation, to effectively limit the number of hypotheses to a few (average of up or average of own regulated genes in tumor vs. normal, in this case), to be quickly tested in the first clinical set available. By using the mouse model as training set and limiting the number of hypotheses, we can help to reduce false positives in clinical setting and speed up the biomarker development.

## Supporting Information

Figure S1 **Mouse tumor signatures predict human patient survival.** Heat maps show the expression of genes that were differentially expressed between tumor and distant normal tissue in the mouse.
(TIF)

Figure S2 **Gene expression signatures in both mouse c-MET liver tumor and human HCC.** **(A) TP53 pathway** signature shown in the same gene order both in mouse (upper panel) and human (lower panel) HCC. Tissue types in mouse: 0 = WT, 1 = DN, 2 = AN, 3 = TU; in human: 0 = AN; 1 = TU. **(B) Wnt signaling pathway** signatures shown in the same gene order both in mouse (upper panel) and human (lower panel) HCC. Tissue types in mouse: 0 = WT, 1 = DN, 2 = AN, 3 = TU; in human: 0 = AN; 1 = TU.
(TIF)

Figure S3 **Genes derived from several mouse models of HCC have predictive power in human HCC.** Log rank test p-value tested in human HCC samples for genes derived by Lee JS compared with permutation.
(TIF)

Table S1 **Mouse tissues used to identify tumor specific signatures.**
(DOCX)

Table S2 **Top 5 GO annotation categories for differentially expressed genes in c-Met tumors.**
(DOCX)

Table S3 **Top 5 GO annotation categories for differentially expressed genes in human HCC tumors.**
(DOCX)

Table S4 **Liver-enriched genes up-regulated in tumors**
(DOCX)

Table S5 **Mouse signatures identify gene sets with predictive power for survival in human samples.**
(DOCX)

Table S6 **Mouse signatures split according to their expression in human samples have prognostic power for disease-free survival.**
(DOCX)

Table S7 **Genes down-regulated in both c-Met tumors and human HCC that have significant predictive power for survival.**
(DOCX)

Table S8 **Gene ontology of different mouse liver tumors versus human HCC gene sets.**
(XLSX)
