# Supplementary figures and images for: Gene Signatures Derived from a c-MET-Driven Liver Cancer Mouse Model Predict Survival of Patients with Hepatocellular Carcinoma
Source: PLoS One. 2011 Sep 16;6(9):e24582. doi: 10.1371/journal.pone.0024582 (PMC3174972; doi:10.1371/journal.pone.0024582)

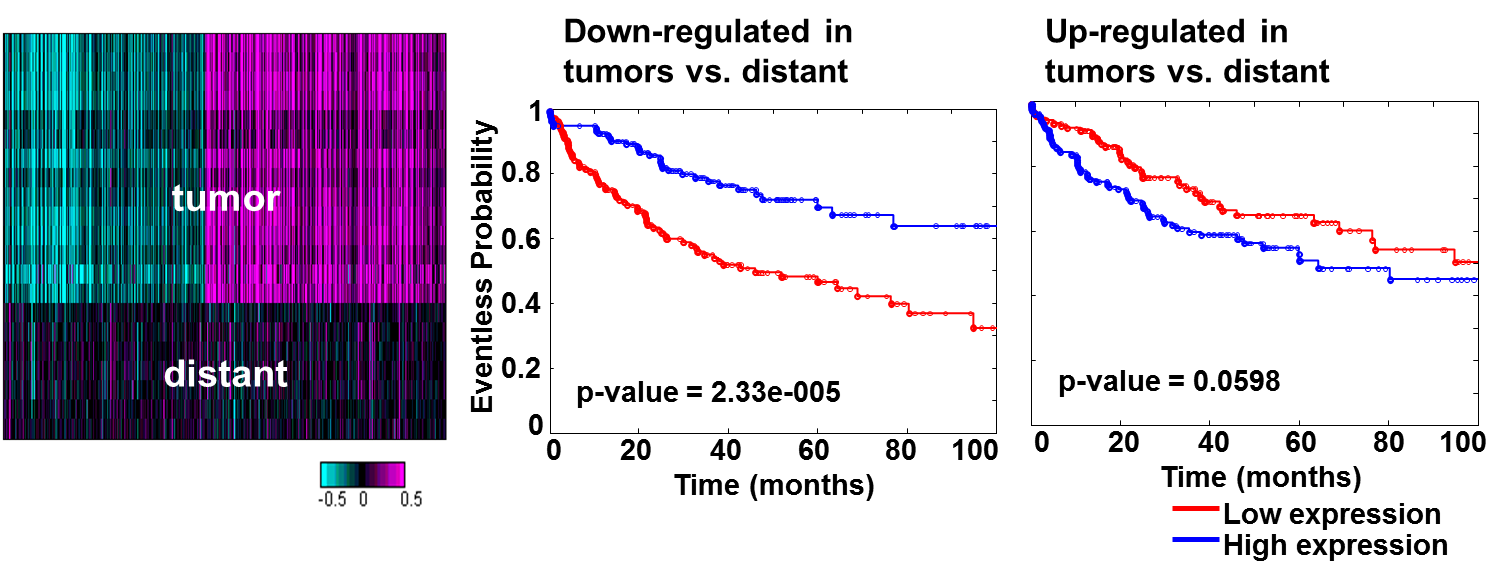

Supplement: Figure S1 — Mouse tumor signatures predict human patient survival. Heat maps show the expression of genes that were differentially expressed between tumor and distant normal tissue in the mouse. (TIF) [file pone.0024582.s001.tif]

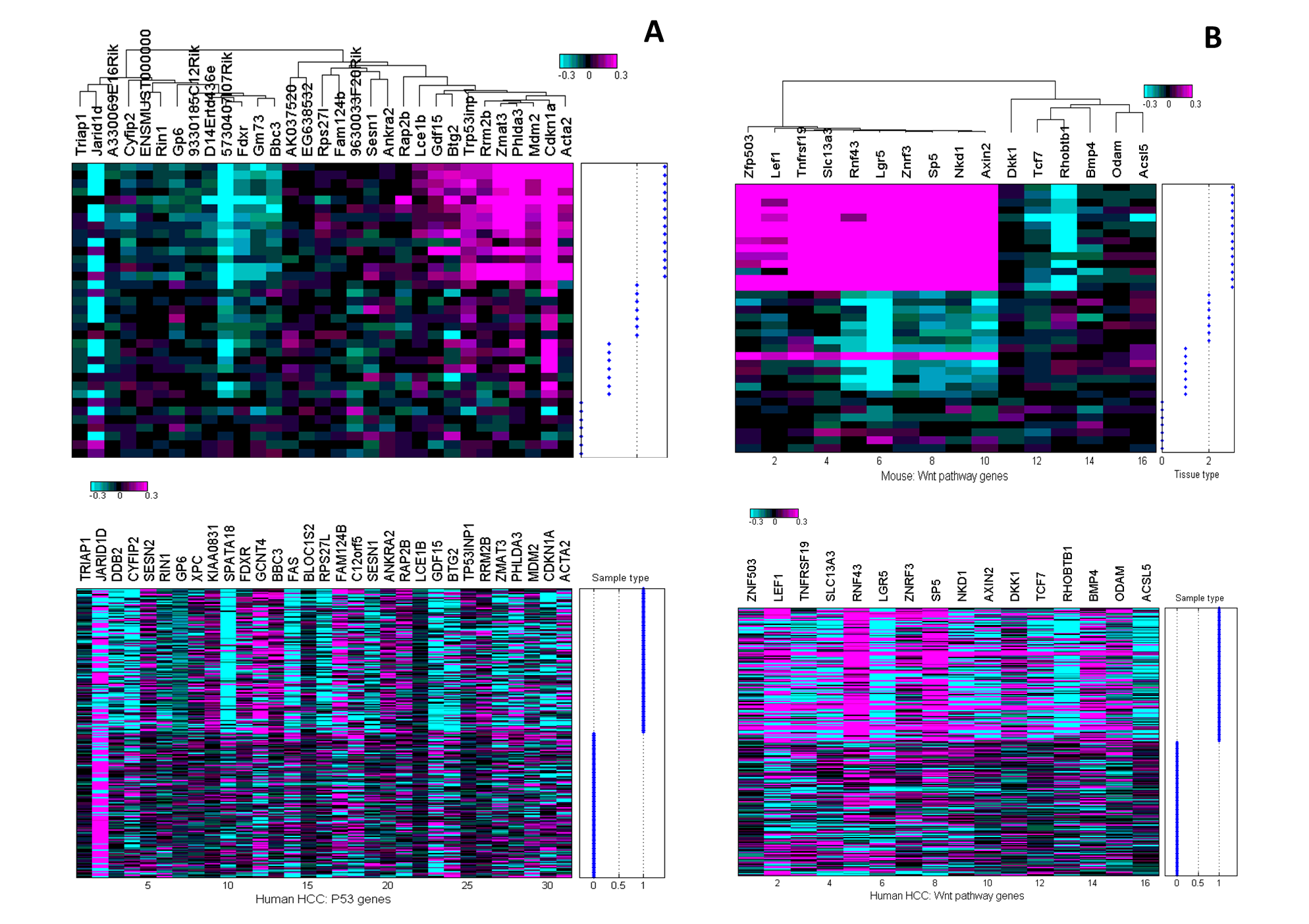

Supplement: Figure S2 — Gene expression signatures in both mouse c-MET liver tumor and human HCC. (A) TP53 pathway signature shown in the same gene order both in mouse (upper panel) and human (lower panel) HCC. Tissue types in mouse: 0 = WT, 1 = DN, 2 = AN, 3 = TU; in human: 0 = AN; 1 = TU. (B) Wnt signaling pathway signatures shown in the same gene order both in mouse (upper panel) and human (lower panel) HCC. Tissue types in mouse: 0 = WT, 1 = DN, 2 = AN, 3 = TU; in human: 0 = AN; 1 = TU. (TIF) [file pone.0024582.s002.tif]

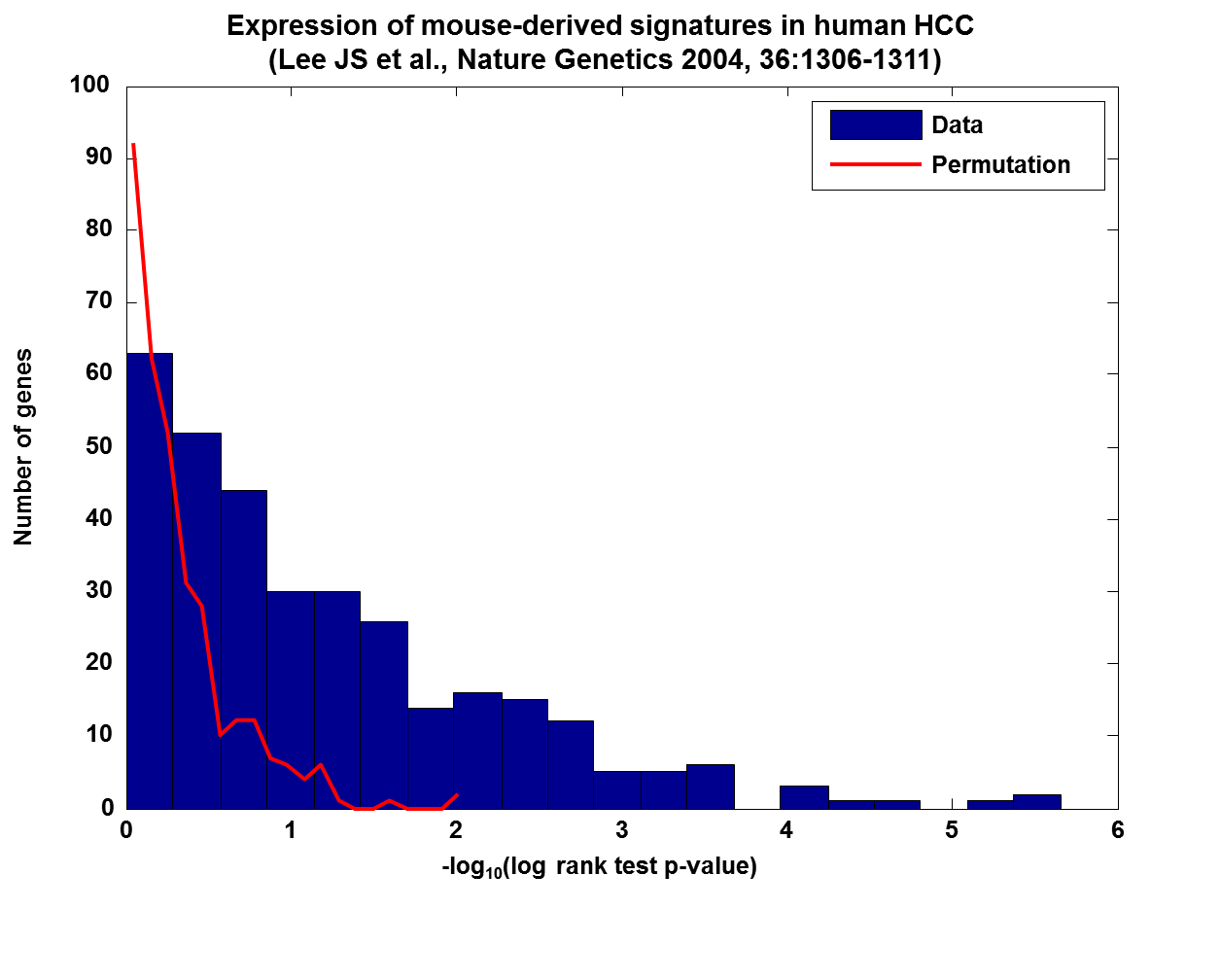

Supplement: Figure S3 — Genes derived from several mouse models of HCC have predictive power in human HCC. Log rank test p-value tested in human HCC samples for genes derived by Lee JS compared with permutation. (TIF) [file pone.0024582.s003.tif]
