# Supplementary material for: Gene Signatures Derived from a c-MET-Driven Liver Cancer Mouse Model Predict Survival of Patients with Hepatocellular Carcinoma
Source: PLoS One. 2011 Sep 16;6(9):e24582. doi: 10.1371/journal.pone.0024582 (PMC3174972; doi:10.1371/journal.pone.0024582)
Supplement: Table S1 — Mouse tissues used to identify tumor specific signatures. (DOCX) [file pone.0024582.s004.docx]

**Table S1. Mouse tissues used to identify tumor specific signatures.**

| **Tissue** | **Strain** | **Transgene present** |
| --- | --- | --- |
| WT | Background | none |
| Control 1 | Parent 1 | LAP-tTa homozygous |
| Control 2 | Parent 2 | TRE-Met homozygous |
| Tumor | c-Met | LAP-tTa + TRE-Met |
| Adjacent | c-Met | LAP-tTa + TRE-Met |
| Distant | c-Met | LAP-tTa + TRE-Met |

All mice were in the FVB genetic background. The c-Met mice carried one copy of the LAP-tTa transgene (the liver-specific LAP promoter driving the Tet-VP16 transactivator) and one copy of the Tre-Met transgene (Tet-operator regulated human c-Met gene). The presence of both transgenes in these mice results in expression of the human c-Met gene specifically in and throughout the liver.
