# Supplementary material for: Gene Signatures Derived from a c-MET-Driven Liver Cancer Mouse Model Predict Survival of Patients with Hepatocellular Carcinoma
Source: PLoS One. 2011 Sep 16;6(9):e24582. doi: 10.1371/journal.pone.0024582 (PMC3174972; doi:10.1371/journal.pone.0024582)
Supplement: Table S2 — Top 5 GO annotation categories for differentially expressed genes in c-Met tumors. (DOCX) [file pone.0024582.s005.docx]

**Table S2. Top 5 GO annotation categories for differentially expressed genes in c-Met tumors**

| **Down-regulated genes** | **Expectation** | **Overlap Gene Count** |
| --- | --- | --- |
| organic acid metabolic process | 4.87E-27 | 202 |
| carboxylic acid metabolic process | 1.11E-26 | 200 |
| monocarboxylic acid metabolic process | 6.06E-18 | 128 |
| cellular amino acid and derivative metabolic process | 2.90E-13 | 96 |
| oxidation reduction | 1.89E-12 | 110 |
|  |  |  |
| **Up-regulated genes** | **Expectation** | **Overlap Gene Count** |
| macromolecular complex subunit organization | 5.91E-15 | 109 |
| cellular macromolecular complex subunit organization | 3.05E-13 | 74 |
| actin cytoskeleton organization | 2.97E-11 | 61 |
| cell division | 1.18E-09 | 67 |
| cellular macromolecular complex assembly | 3.23E-09 | 56 |
