# Supplementary material for: Gene Signatures Derived from a c-MET-Driven Liver Cancer Mouse Model Predict Survival of Patients with Hepatocellular Carcinoma
Source: PLoS One. 2011 Sep 16;6(9):e24582. doi: 10.1371/journal.pone.0024582 (PMC3174972; doi:10.1371/journal.pone.0024582)
Supplement: Table S3 — Top 5 GO annotation categories for differentially expressed genes in human HCC tumors. (DOCX) [file pone.0024582.s006.docx]

**Table S3: Top 5 GO annotation categories for differentially expressed genes in human HCC tumors**

| **Down-regulated genes** | **Expectation** |
| --- | --- |
| Oxidoreductase | 1.41E-23 |
| Oxygenase | 2.54E-20 |
| Oxidoreductase activity | 9.78E-19 |
| carboxylic acid metabolic process | 2.95E-18 |
| organic acid metabolic process | 4.11E-18 |
|  |  |
| **Up-regulated genes** | **Expectation** |
| mitotic cell cycle | 2.27E-39 |
| cell cycle phase | 2.79E-39 |
| mitosis | 5.09E-37 |
| M phase of mitotic cell cycle | 5.76E-37 |
| M phase | 4.51E-36 |
