# Supplementary material for: Gene Signatures Derived from a c-MET-Driven Liver Cancer Mouse Model Predict Survival of Patients with Hepatocellular Carcinoma
Source: PLoS One. 2011 Sep 16;6(9):e24582. doi: 10.1371/journal.pone.0024582 (PMC3174972; doi:10.1371/journal.pone.0024582)
Supplement: Table S4 — Liver-enriched genes up-regulated in tumors (DOCX) [file pone.0024582.s007.docx]

**Table S4. Liver-enriched genes up-regulated in tumors**

| **Figure 1C, box 1** | | **Figure 1C, box 2** | |
| --- | --- | --- | --- |
| **Fn1** | Lman2l | |  |
| Cyp3a16 | Bst2 | |  |
| Cyp2a4 | Abca8b | |  |
| **Serpina6** | **F5** | |  |
| Cyp2c69 | **Afm** | |  |
| **Cfhr1** | **Habp2** | |  |
| Ctsh | Htatip2 | |  |
| **Sepp1** | **Igfbp1** | |  |
| **Apcs** | 2900006A08Rik | |  |
| **Fgl1** | Lgtn | |  |
| **Cp** | 8030488J09Rik | |  |
| Trf | Avpr1a | |  |
| Errfi1 | Aqp9 | |  |
| Ccdc134 | **Lect2** | |  |
| Rnase4 | Gulo | |  |
| 5033414K04Rik | Akr1c20 | |  |
|  | Glud1 | |  |
|  | 1810021J13Rik | |  |
|  | **Nrn1** | |  |

Note: In bold are secreted proteins that may be potential serum biomarkers of tumor formation in the mouse model.
