# Supplementary material for: Gene Signatures Derived from a c-MET-Driven Liver Cancer Mouse Model Predict Survival of Patients with Hepatocellular Carcinoma
Source: PLoS One. 2011 Sep 16;6(9):e24582. doi: 10.1371/journal.pone.0024582 (PMC3174972; doi:10.1371/journal.pone.0024582)
Supplement: Table S5 — Mouse signatures identify gene sets with predictive power for survival in human samples. (DOCX) [file pone.0024582.s008.docx]

**Table S5. Mouse signatures identify gene sets with predictive power for survival in human samples.**

|  | **Tumor** | | |
| --- | --- | --- | --- |
|  | down | | up |
| **WT** | 9.5 x 10^-6^ | 0.07 | |
| **adjacent** | 2.0 x 10^-5^ | 0.10 | |
| **distant** | 2.3 x 10^-5^ | 0.06 | |

Pair-wise comparison between tumors and WT, adjacent or distant samples identified expression signatures for genes that were either down-regulated or up-regulated in the tumors. The p-values for the ability of these signatures to predict survival in human patients is indicated based on KM curves in Figure 4.
