# Supplementary material for: Gene Signatures Derived from a c-MET-Driven Liver Cancer Mouse Model Predict Survival of Patients with Hepatocellular Carcinoma
Source: PLoS One. 2011 Sep 16;6(9):e24582. doi: 10.1371/journal.pone.0024582 (PMC3174972; doi:10.1371/journal.pone.0024582)
Supplement: Table S6 — Mouse signatures split according to their expression in human samples have prognostic power for disease-free survival. (DOCX) [file pone.0024582.s009.docx]

**Table S6. Mouse signatures split according to their expression in human samples have prognostic power for disease-free survival**

| **Mouse** | **Down in tumor** | | **Up in tumor** | |
| --- | --- | --- | --- | --- |
| **Human** | **down** | **up** | **down** | **up** |
| **Tumor compared to** |  |  |  |  |
| WT | 1.0 x 10^-5^ | 0.18 | 0.09 | 0.010 |
| adjacent | 1.1 x 10^-5^ | 0.30 | 0.27 | 0.0003 |
| distant | 3.7 x 10^-5^ | 0.76 | 0.33 | 0.0023 |

Mouse gene expression signatures were split according to the expression of the genes in human tissues. The ability of each of the gene sets to predict disease-free survival in human samples was assessed using KM plots. The p-values for prognosis were calculated and are indicated in the table.
