# Supplementary material for: Gene Signatures Derived from a c-MET-Driven Liver Cancer Mouse Model Predict Survival of Patients with Hepatocellular Carcinoma
Source: PLoS One. 2011 Sep 16;6(9):e24582. doi: 10.1371/journal.pone.0024582 (PMC3174972; doi:10.1371/journal.pone.0024582)
Supplement: Table S7 — Genes down-regulated in both c-Met tumors and human HCC that have significant predictive power for survival. (DOCX) [file pone.0024582.s010.docx]

**Table S7. Genes down-regulated in both c-Met tumors and human HCC that have significant predictive power for survival**

| **Accession #** | **Gene name** |
| --- | --- |
| NM_182662 | AADAT |
| NM_005763 | AASS |
| NM_020686 | ABAT |
| NM_212533 | ABCA2 |
| NM_003786 | ABCC3 |
| NM_016428 | ABI3 |
| NM_014945 | ABLIM3 |
| NM_001093 | ACACB |
| NM_001609 | ACADSB |
| NM_014716 | ACAP1 |
| NM_024722 | ACBD4 |
|  | ACOX3 |
|  | ACP5 |
| NM_000666 | ACY1 |
| NM_139027 | ADAMTS13 |
| NM_207517 | ADAMTSL3 |
| NM_024551 | ADIPOR2 |
| NM_172197 | AGER |
| NM_001012727 | AGPAT2 |
| NM_000030 | AGXT |
| NM_031279 | AGXT2L1 |
| NM_016282 | AK3 |
| NM_001353 | AKR1C1 |
| NM_005989 | AKR1D1 |
| NM_000689 | ALDH1A1 |
| NM_000692 | ALDH1B1 |
| NM_012190 | ALDH1L1 |
| NM_152435 | AMDHD1 |
| NM_031917 | ANGPTL6 |
| NM_182703 | ANKDD1A |
| NM_198401 | ANKRD46 |
| NM_001012302 | ANO9 |
| NM_001163 | APBA1 |
| NM_052968 | APOA5 |
| NM_145641 | APOL3 |
| NM_030641 | APOL6 |
| NM_198098 | AQP1 |
| NM_173039 | AQP11 |
| NM_000045 | ARG1 |
| NM_015071 | ARHGAP26 |
| NM_032496 | ARHGAP9 |
| NM_005737 | ARL4C |
| NM_001661 | ARL4D |
| NM_001025604 | ARRDC2 |
| NM_183376 | ARRDC4 |
| NM_000049 | ASPA |
| NM_020437 | ASPHD2 |
| NM_000050 | ASS1 |
| NM_018036 | ATG2B |
| NM_020453 | ATP10D |
| NM_207303 | ATRNL1 |
| NM_012105 | BACE2 |
| NM_004874 | BAG4 |
| NM_017935 | BANK1 |
| NM_020139 | BDH2 |
| NM_001713 | BHMT |
| NM_017614 | BHMT2 |
| NM_006129 | BMP1 |
| NM_001200 | BMP2 |
| NM_001718 | BMP6 |
| NM_033271 | BTBD6 |
| NM_000060 | BTD |
| NM_014670 | BZW1 |
| NM_198472 | C10orf125 |
|  | C10orf26 |
| NM_019021 | C11orf71 |
| NM_144581 | C14orf149 |
| NM_207117 | C14orf68 |
| NM_024052 | C17orf39 |
| NM_020233 | C17orf48 |
| NM_207103 | C17orf87 |
| NM_024805 | C18orf22 |
| NM_178837 | C19orf51 |
| NM_018381 | C19orf66 |
| NM_001010980 | C1orf130 |
| NM_138428 | C1orf212 |
| NM_001733 | C1R |
| NM_024520 | C2orf47 |
| NM_001039717 | C4orf29 |
| NM_000066 | C8B |
| NM_020130 | C8orf4 |
| NM_001737 | C9 |
| NM_203403 | C9orf150 |
| NM_001039395 | C9orf68 |
| NM_153045 | C9orf91 |
| NM_017881 | C9orf95 |
| NM_001738 | CA1 |
| NM_001217 | CA11 |
| NM_031468 | CALN1 |
| NM_015215 | CAMTA1 |
| NM_033340 | CASP7 |
| NM_001752 | CAT |
| NM_000071 | CBS |
| NM_138803 | CCDC148 |
| NM_016557 | CCRL1 |
| NM_007053 | CD160 |
| NM_004244 | CD163 |
| NM_015717 | CD207 |
| NM_021155 | CD209 |
| NM_198053 | CD247 |
| NM_181449 | CD300E |
| NM_001775 | CD38 |
| NM_000574 | CD55 |
| NM_005894 | CD5L |
| NM_001251 | CD68 |
| NM_001783 | CD79A |
| NM_004933 | CDH15 |
| NM_177980 | CDH26 |
| NM_002483 | CEACAM6 |
| NM_016174 | CERCAM |
| NM_003869 | CES2 |
| NM_001928 | CFD |
| NM_000186 | CFH |
| NM_005666 | CFHR2 |
| NM_000744 | CHRNA4 |
|  | CLEC1B |
| NM_173535 | CLEC4F |
| NM_198492 | CLEC4G |
| NM_206808 | CLYBL |
|  | CMAH |
| NM_153610 | CMYA5 |
| NM_001841 | CNR2 |
| NM_080679 | COL11A2 |
| NM_080811 | COL13A1 |
| NM_006438 | COLEC10 |
| NM_199235 | COLEC11 |
| NM_006091 | CORO2B |
| NM_181654 | CPLX4 |
| NM_001875 | CPS1 |
| NM_000098 | CPT2 |
| NM_001310 | CREBL2 |
| NM_015974 | CRYL1 |
| NM_005213 | CSTA |
| NM_001902 | CTH |
| NM_001907 | CTRL |
| NM_001814 | CTSC |
| NM_015267 | CUX2 |
| NM_000609 | CXCL12 |
| NM_002089 | CXCL2 |
| NM_144611 | CYB5D2 |
| NM_000104 | CYP1B1 |
|  | CYP2B7P1 |
| NM_000769 | CYP2C19 |
| NM_000775 | CYP2J2 |
| NM_017460 | CYP3A4 |
| NM_004391 | CYP8B1 |
| NM_152783 | D2HGDH |
| NM_014992 | DAAM1 |
| NM_145056 | DACT3 |
| NM_004938 | DAPK1 |
| NM_002036 | DARC |
| NM_014026 | DCPS |
| NM_016286 | DCXR |
| NM_020664 | DECR2 |
| NM_024898 | DENND1C |
| NM_001345 | DGKA |
| NM_018706 | DHTKD1 |
| NM_024119 | DHX58 |
| NM_000792 | DIO1 |
| NM_001362 | DIO3 |
| NM_001037954 | DIXDC1 |
| NM_021120 | DLG3 |
| NM_005618 | DLL1 |
| NM_020877 | DNAH2 |
| NM_001539 | DNAJA1 |
| NM_013238 | DNAJC15 |
| NM_004944 | DNASE1L3 |
| NM_203447 | DOCK8 |
| NM_012074 | DPF3 |
| NM_020868 | DPP10 |
| NM_000110 | DPYD |
|  | DTX4 |
| NM_006519 | DYNLT1 |
| NM_032565 | EBPL |
| NM_022336 | EDAR |
| NM_001402 | EEF1A1 |
| NM_004430 | EGR3 |
| NM_014600 | EHD3 |
| NM_012307 | EPB41L3 |
| NM_004445 | EPHB6 |
| NM_000121 | EPOR |
| NM_004453 | ETFDH |
| NM_005244 | EYA2 |
| NM_000504 | F10 |
| NM_005242 | F2RL1 |
| NM_004101 | F2RL2 |
| NM_000132 | F8 |
| NM_001018104 | FAHD1 |
| NM_012306 | FAIM2 |
| NM_005449 | FAIM3 |
| NM_014883 | FAM13A1 |
|  | FAM155A |
| NM_207446 | FAM174B |
| NM_017709 | FAM46C |
| NM_024792 | FAM57A |
| NM_001006605 | FAM69A |
| NM_000138 | FBN1 |
| NM_000507 | FBP1 |
| NM_015837 | FCN2 |
| NM_003665 | FCN3 |
| NM_032738 | FCRLA |
| NM_005103 | FEZ1 |
| NM_000508 | FGA |
| NM_023105 | FGFR1 |
| NM_022970 | FGFR2 |
| NM_005248 | FGR |
| NM_025135 | FHOD3 |
| NM_004117 | FKBP5 |
| NM_022823 | FNDC4 |
| NM_153756 | FNDC5 |
| NM_002029 | FPR1 |
| NM_001005738 | FPR2 |
| NM_023037 | FRY |
| NM_006657 | FTCD |
| NM_000147 | FUCA1 |
| NM_025129 | FUZ |
| NM_031866 | FZD8 |
| NM_000151 | G6PC |
| NM_138801 | GALM |
| NM_002048 | GAS1 |
| NM_002052 | GATA4 |
| NM_000158 | GBE1 |
| NM_000159 | GCDH |
| NM_001024024 | GCH1 |
| NM_015721 | GEMIN4 |
| NM_153338 | GGT6 |
| NM_000163 | GHR |
| NM_024711 | GIMAP6 |
| NM_002060 | GJA4 |
| NM_004004 | GJB2 |
| NM_015554 | GLCE |
| NM_000170 | GLDC |
| NM_005270 | GLI2 |
| NM_013267 | GLS2 |
| NM_201648 | GLYAT |
| NM_018960 | GNMT |
| NM_001010917 | GOLGA7B |
| NM_002079 | GOT1 |
| NM_000173 | GP1BA |
|  | GPNMB |
| NM_032777 | GPR124 |
|  | GPR18 |
| NM_133443 | GPT2 |
| NM_025196 | GRPEL1 |
| NM_000853 | GSTT1 |
| NM_002101 | GYPC |
| NM_033423 | GZMH |
| NM_012260 | HACL1 |
| NM_005326 | HAGH |
| NM_021175 | HAMP |
| NM_032782 | HAVCR2 |
|  | hCG_20857 |
| NM_144657 | HDX |
| NM_015987 | HEBP1 |
| NM_014571 | HEYL |
| NM_152419 | HGSNAT |
| NM_032593 | HINT2 |
| NM_006895 | HNMT |
| NM_199331 | HOMER2 |
| NM_018952 | HOXB6 |
| NM_002152 | HRC |
| NM_005836 | HRSP12 |
| NM_181755 | HSD11B1 |
| NM_178135 | HSD17B13 |
| NM_003725 | HSD17B6 |
| NM_012092 | ICOS |
| NM_001546 | ID4 |
| NM_006820 | IFI44L |
| NM_207585 | IFNAR2 |
| NM_000618 | IGF1 |
| NM_004970 | IGFALS |
| NM_000597 | IGFBP2 |
| NM_001560 | IL13RA1 |
| NM_000640 | IL13RA2 |
| NM_002182 | IL1RAP |
| NM_144717 | IL20RB |
| NM_006850 | IL24 |
| NM_194294 | INDOL1 |
| NM_198336 | INSIG1 |
| NM_002199 | IRF2 |
| NM_024710 | ISOC2 |
| NM_031483 | ITCH |
| NM_002215 | ITIH1 |
| NM_002217 | ITIH3 |
| NM_021219 | JAM2 |
| NM_001024660 | KALRN |
| NM_003884 | KAT2B |
| NM_002232 | KCNA3 |
| NM_003740 | KCNK5 |
| NM_024076 | KCTD15 |
| NM_002253 | KDR |
| NM_006488 | KHK |
| NM_014804 | KIAA0753 |
| NM_017644 | KLHL24 |
| NM_006611 | KLRA1 |
| NM_007360 | KLRK1 |
| NM_003937 | KYNU |
| NM_005559 | LAMA1 |
| NM_004139 | LBP |
| NM_020997 | LEFTY1 |
| NM_017980 | LIMS2 |
| NM_004664 | LIN7A |
| NM_001010939 | LIPJ |
| NM_014646 | LPIN2 |
| NM_004744 | LRAT |
| NM_002336 | LRP6 |
| NM_144598 | LRRC28 |
| NM_022143 | LRRC4 |
| NM_020929 | LRRC4C |
| NM_178011 | LRRTM3 |
| NM_007080 | LSM6 |
| NM_181705 | LYRM7 |
| NM_014067 | MACROD1 |
|  | MAGIX |
| NM_153267 | MAMDC2 |
| NM_001039580 | MAP9 |
| NM_139049 | MAPK8 |
| NM_014268 | MAPRE2 |
| NM_006770 | MARCO |
| NM_001879 | MASP1 |
| NM_006610 | MASP2 |
| NM_000429 | MAT1A |
|  | MCC |
| NM_022132 | MCCC2 |
| NM_032601 | MCEE |
| NM_001039845 | MDH1B |
| NM_002399 | MEIS2 |
| NM_152637 | METTL7B |
| NM_032793 | MFSD2 |
| NM_181644 | MFSD4 |
| NM_032718 | MFSD9 |
| NM_004668 | MGAM |
| NM_001003794 | MGLL |
| NM_002412 | MGMT |
| NM_012329 | MMD |
| NM_002430 | MN1 |
| NM_022746 | MOSC1 |
| NM_004870 | MPDU1 |
| NM_003829 | MPDZ |
|  | MPPED1 |
| NM_152866 | MS4A1 |
| NM_005956 | MTHFD1 |
| NM_006441 | MTHFS |
|  | MTUS1 |
|  | MYH11 |
| NM_013262 | MYLIP |
| NM_015011 | MYO16 |
|  | MYO3B |
| NM_133371 | MYOZ3 |
| NM_052818 | N4BP2L1 |
| NM_000263 | NAGLU |
| NM_024678 | NARS2 |
| NM_000662 | NAT1 |
| NM_005000 | NDUFA5 |
| NM_016013 | NDUFAF1 |
| NM_024608 | NEIL1 |
| NM_024800 | NEK11 |
| NM_005595 | NFIA |
| NM_005596 | NFIB |
| NM_002507 | NGFR |
| NM_178844 | NLRC3 |
| NM_006169 | NNMT |
| NM_022162 | NOD2 |
|  | NPW |
| NM_000904 | NQO2 |
| NM_001010848 | NRG3 |
| NM_138573 | NRG4 |
| NM_021229 | NTN4 |
|  | NTRK2 |
| NM_017681 | NUP62CL |
| NM_178507 | OAF |
| NM_003733 | OASL |
| NM_024578 | OCEL1 |
| NM_152635 | OIT3 |
| NM_198474 | OLFML1 |
| NM_000531 | OTC |
| NM_000915 | OXT |
| NM_002563 | P2RY1 |
| NM_176894 | P2RY13 |
| NM_148977 | PANK1 |
|  | PAOX |
| NM_001040202 | PAQR3 |
| NM_022129 | PBLD |
| NM_000282 | PCCA |
| NM_020403 | PCDH9 |
| NM_002591 | PCK1 |
| NM_002598 | PDCD2 |
| NM_000922 | PDE3B |
| NM_018945 | PDE7B |
|  | PDE9A |
| NM_006810 | PDIA5 |
| NM_006210 | PEG3 |
| NM_020651 | PELI1 |
| NM_000288 | PEX7 |
| NM_173582 | PGM2L1 |
| NM_006320 | PGRMC2 |
| NM_006623 | PHGDH |
|  | PHYHD1 |
| NM_004278 | PIGL |
| NM_002644 | PIGR |
| NM_000930 | PLAT |
| NM_015184 | PLCL2 |
| NM_021200 | PLEKHB1 |
| NM_032812 | PLXDC2 |
| NM_025225 | PNPLA3 |
| NM_203453 | PPAPDC2 |
| NM_152542 | PPM1K |
| NM_024607 | PPP1R3B |
| NM_181699 | PPP2R1B |
| NM_000944 | PPP3CA |
| NM_020820 | PREX1 |
| NM_002728 | PRG2 |
| NM_153026 | PRICKLE1 |
| NM_016203 | PRKAG2 |
| NM_005400 | PRKCE |
| NM_006257 | PRKCQ |
| NM_002764 | PRPS1 |
| NM_006799 | PRSS21 |
| NM_024754 | PTCD2 |
| NM_000314 | PTEN |
| NM_000954 | PTGDS |
| NM_080591 | PTGS1 |
| NM_000963 | PTGS2 |
| NM_000316 | PTHR1 |
|  | PTPRB |
| NM_002854 | PVALB |
| NM_015480 | PVRL3 |
| NM_018663 | PXMP2 |
| NM_002863 | PYGL |
| NM_000320 | QDPR |
| NM_001004128 | QSOX1 |
| NM_183235 | RAB27A |
| NM_006834 | RAB32 |
| NM_022337 | RAB38 |
| NM_145000 | RANBP3L |
| NM_002885 | RAP1GAP |
| NM_152545 | RASGEF1B |
| NM_000321 | RB1 |
| NM_144979 | RBM46 |
| NM_001268 | RCBTB2 |
| NM_017750 | RETSAT |
| NM_020695 | REXO1 |
| NM_001029875 | RGS7BP |
| NM_001032380 | RHOBTB1 |
| NM_004292 | RIN1 |
| NM_005440 | RND2 |
| NM_017831 | RNF125 |
| NM_018434 | RNF130 |
| NM_003799 | RNMT |
| NM_031916 | ROPN1L |
| NM_001033002 | RPAIN |
| NM_020366 | RPGRIP1 |
| NM_000987 | RPL26 |
| NM_000995 | RPL34 |
| NM_002953 | RPS6KA1 |
| NM_001006932 | RPS6KA2 |
| NM_032784 | RSPO3 |
| NM_002957 | RXRA |
| NM_001024210 | S100A13 |
| NM_030760 | S1PR5 |
|  | SAMD12 |
| NM_015474 | SAMHD1 |
| NM_007101 | SARDH |
| NM_006918 | SC5DL |
| NM_001038 | SCNN1A |
| NM_014766 | SCRN1 |
| NM_002999 | SDC4 |
| NM_003000 | SDHB |
| NM_006843 | SDS |
| NM_138432 | SDSL |
| NM_174977 | SEC14L4 |
| NM_020796 | SEMA6A |
| NM_052838 | SEPT1 |
| NM_016332 | SEPX1 |
| NM_001085 | SERPINA3 |
| NM_000624 | SERPINA5 |
| NM_144579 | SFXN5 |
| NM_005627 | SGK1 |
| NM_003026 | SH3GL2 |
| NM_004169 | SHMT1 |
| NM_013276 | SHPK |
| NM_023068 | SIGLEC1 |
| NM_052884 | SIGLEC11 |
|  | SIRT3 |
| NM_003726 | SKAP1 |
| NM_003049 | SLC10A1 |
| NM_177550 | SLC13A5 |
| NM_004694 | SLC16A6 |
| NM_194298 | SLC16A9 |
| NM_005835 | SLC17A2 |
| NM_004171 | SLC1A2 |
| NM_006749 | SLC20A2 |
| NM_153320 | SLC22A7 |
| NM_014252 | SLC25A15 |
| NM_001006641 | SLC25A25 |
| NM_012254 | SLC27A5 |
| NM_000340 | SLC2A2 |
| NM_001001290 | SLC2A9 |
| NM_021194 | SLC30A1 |
| NM_001467 | SLC37A4 |
| NM_018018 | SLC38A4 |
| NM_022154 | SLC39A8 |
| NM_033102 | SLC45A3 |
| NM_018242 | SLC47A1 |
| NM_016615 | SLC6A13 |
| NM_003046 | SLC7A2 |
| NM_007256 | SLCO2B1 |
| NM_005905 | SMAD9 |
| NM_139045 | SMARCA2 |
| NM_006714 | SMPDL3A |
| NM_001080437 | SNED1 |
|  | SNORA75 |
| NM_003877 | SOCS2 |
|  | SOD2 |
| NM_003102 | SOD3 |
| NM_015087 | SPG20 |
|  | SPNS2 |
| NM_014767 | SPOCK2 |
| NM_207344 | SPRYD4 |
| NM_021947 | SRR |
|  | SS18 |
| NM_006278 | ST3GAL4 |
| NM_003896 | ST3GAL5 |
| NM_018412 | ST7 |
| NM_005668 | ST8SIA4 |
| NM_012108 | STAP1 |
| NM_033050 | SUCNR1 |
| NM_017503 | SURF2 |
|  | SYDE2 |
| NM_182961 | SYNE1 |
| NM_054114 | TAGAP |
| NM_152334 | TARSL2 |
| NM_000353 | TAT |
| NM_000355 | TCN2 |
| NM_005651 | TDO2 |
| NM_198795 | TDRD1 |
| NM_000459 | TEK |
| NM_031275 | TEX12 |
| NM_003226 | TFF3 |
| NM_003227 | TFR2 |
| NM_199003 | THAP1 |
| NM_000461 | THRB |
| NM_003251 | THRSP |
| NM_138379 | TIMD4 |
| NM_020698 | TMCC3 |
|  | TMEM100 |
| NM_025124 | TMEM134 |
| NM_018295 | TMEM140 |
| NM_001100389 | TMEM192 |
| NM_018202 | TMEM57 |
| NM_003275 | TMOD1 |
| NM_153609 | TMPRSS6 |
| NM_207381 | TNFAIP8L3 |
| NM_003810 | TNFSF10 |
| NM_015028 | TNIK |
|  | TOX2 |
| NM_001003397 | TPD52L1 |
| NM_003596 | TPST1 |
| NM_001042646 | TRAK1 |
| NM_015271 | TRIM2 |
| NM_004615 | TSPAN7 |
| NM_000370 | TTPA |
| NM_030773 | TUBB1 |
| NM_032525 | TUBB6 |
|  | TXNRD3 |
| NM_152376 | UBXN10 |
| NM_001074 | UGT2B7 |
| NM_000373 | UMPS |
| NM_173355 | UPP2 |
| NM_006003 | UQCRFS1 |
| NM_144639 | UROC1 |
| NM_004624 | VIPR1 |
| NM_003382 | VIPR2 |
| NM_015378 | VPS13D |
|  | VSIG4 |
| NM_021197 | WFDC1 |
| NM_006103 | WFDC2 |
| NM_003391 | WNT2 |
| NM_003396 | WNT9B |
| NM_175569 | XG |
| NM_001005404 | YPEL2 |
| NM_175907 | ZADH2 |
| NM_006006 | ZBTB16 |
| NM_153367 | ZCCHC24 |
|  | ZMAT1 |
| NM_032265 | ZMYND15 |
| NM_025169 | ZNF167 |
| NM_152287 | ZNF276 |
| NM_018660 | ZNF395 |
| NM_178558 | ZNF680 |
| NM_001080464 |  |
| NM_015595 |  |
| NM_016459 |  |
| NM_032435 |  |
| NM_213726 |  |
